# Nodal Promotes the Migration and Invasion of Bladder Cancer Cells via Regulation of Snail: Erratum

**DOI:** 10.7150/jca.71011

**Published:** 2022-02-17

**Authors:** Wenwei Chen, Tao Jiang, Houping Mao, Rui Gao, Xingjian Gao, Yanfeng He, Hua Zhang, Qin Chen

**Affiliations:** Department of Urology, The First Affiliated Hospital of Fujian Medical University, Fuzhou 350005, China

In our paper 1, the western blot band for GAPDH (Figure 6D) was used wrong. We reperformed this experiment and provided corrected Figure 6D. The correction made in this erratum does not affect the counting results and original conclusions. We apologize for the error and for any inconvenience that may cause to the readers and the editors of this journal.

## Figures and Tables

**Figure 6:**
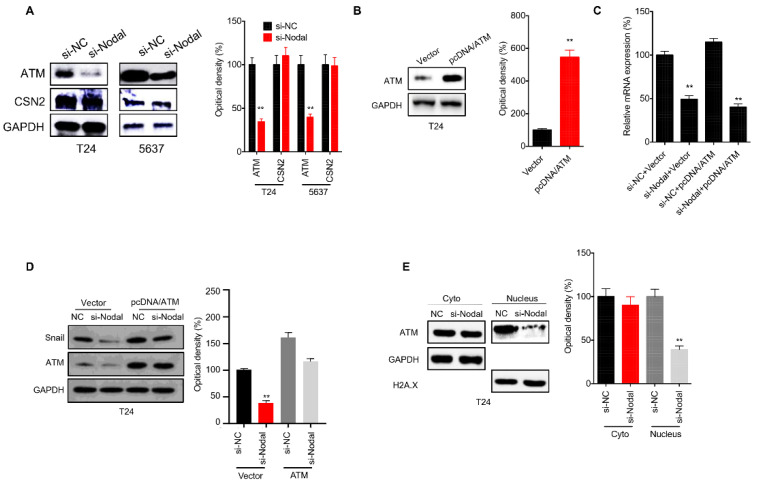
***ATM is involved in Nodal induced stabilization of Snail in bladder cancer cells***. (A) T24 or 5637 cells were transfected with si-NC or si-Nodal for 24 h, the expression of ATM and CSN2 was checked by western blot analysis; (B) T24 cells were transfected with vector control or pcDNA/ATM for 24 h; T24 cells were transfected with si-NC, si-Nodal, vector control, or pcDNA/ATM for 24 h, the mRNA (C) or protein (D) expression of Snail was checked by qRT-PCR and western blot analysis, respectively. (E) T24 cells were transfected with si-NC or si-Nodal for 24 h, the expression of ATM in cytoplasm and nucleus was checked by western blot analysis. Data are presented as means ± SD of three independent experiments. ** p<0.01 compared with control.
